# Diagnostic value of circulating lncRNAs for gastric cancer: A systematic review and meta-analysis

**DOI:** 10.3389/fonc.2022.1058028

**Published:** 2022-12-06

**Authors:** Jian Li, Yanyan Zhang, Qingyu Xu, Yaqiong Zhang, Songhua Bei, Ying Ding, Xiaohong Zhang, Li Feng

**Affiliations:** Endoscopy Center, Minhang Hospital, Fudan University, Shanghai, China

**Keywords:** gastric cancer, lncRNA, diagnosis, systematic review, meta - analysis

## Abstract

**Objective:**

With the prevalence of next-generation sequencing (NGS) technology, a large number of long non-coding RNAs (lncRNAs) have attracted tremendous attention and have been the topic of extensive research on gastric cancer (GC). It was revealed that lncRNAs not only participate in the transduction of various signaling pathways, thus influencing GC genesis and development, but also have the potential for GC diagnosis. Therefore, we aimed to conduct a meta-analysis of previous studies on GC.

**Materials and methods:**

An electronic search was made before August 2021 on databases including PubMed, Embase, and Web of Science. Relevant articles that compare lncRNA expression in GC patients and healthy controls were summarized. We conducted a meta-analysis with the objective of evaluating the ability of lncRNAs in diagnosing GC.

**Results:**

A total of 40 original research studies including 6,772 participants were discussed in this meta-analysis. The overall sensitivity, specificity, and the area under the curve (AUC) were 0.78 (95% CI: 0.75–0.81), 0.79 (95% CI: 0.74–0.83), and 0.85 (95% CI: 0.81–0.87), respectively. The value of pooled diagnostic odds ratios (DORs) was 13.00 (95% CI: 10.00–17.00).

**Conclusions:**

This meta-analysis revealed that serum or plasma lncRNAs have high sensitivity and specificity, which makes lncRNAs clinically feasible in diagnosing GC. The results from this meta-analysis demonstrated that peripheral blood lncRNAs may become novel noninvasive biomarkers in the foreseeable future. At the same time, it should be noted that a greater number of blood samples and more evidence from rigorous multicenter clinical studies are necessary to justify their applicability as cancer biomarkers.

## Introduction

Cancer is the leading cause of death and is a significant obstacle in the pursuit of a higher life expectancy worldwide (1). Unfortunately, the incidence and mortality of cancer are growing rapidly. Gastric cancer (GC) is an important malignant tumor in the digestive tract. According to the latest data, in 2020 alone, there are over 1 million new patients diagnosed with GC and about 769,000 cases die from it (2). It is widely accepted that chronic *Helicobacter pylori* (*H. pylori*) infection is the primary cause of GC (3, 4), and the International Agency for Research on Cancer (IARC) cited *H. pylori* as a group 1 carcinogen (5, 6).

The present treatment strategy for early GC usually depends on endoscopic surgery, while for advanced GC, the treatment methods include surgery, chemotherapy, and immunotherapy (7). Although progress has been achieved in GC treatment, challenges in terms of diagnosis remain. By the time symptoms appear in patients, most of them have already been diagnosed with an advanced stage of cancer (8), which seriously affects their prognosis and 5-year survival rate (9). Currently, gastrointestinal endoscopy operation together with biopsy is the main approach to identifying GC lesions, but detecting small lesions proved to be difficult because of the limited experience of endoscopists (5). In addition, patients find it difficult to undergo endoscopy because it is an invasive procedure and causes discomfort. Consequently, noninvasive biomarkers tend to be a better choice to solve this difficulty. From the traditional point of view, biomarkers in detecting GC can be classified from serum and gastric juice: serum biomarkers included carcinoembryonic antigen (CEA), carbohydrate antigen 199 (CA199), carbohydrate antigen 724 (CA724), and pepsinogen (PG) (10). Gastric juice biomarkers included CA724, CEA, CA199, CA242, and α1-antitrypsin (11, 12). However, the low sensitivity and specificity of these biomarkers in detecting GC limit their further application (13). Therefore, exploring novel biomarkers is of great importance in GC diagnosis.

With the increasing popularity of NGS applications, a large number of studies have been conducted to identify the role of lncRNAs in various tumors over several decades. Long non-coding RNAs, a class of non-coding RNA molecules with a length of more than 200 nt and lacking open reading frames, are closely associated with tumor invasion (14), metastasis, and drug resistance (15) of GC through multiple pathways. Moreover, studies also evaluated the diagnostic value of lncRNAs in distinguishing GC patients from healthy volunteers. These studies have demonstrated that the expression of lncRNAs could be a novel biomarker in screening GC due to their high sensitivity and specificity. Therefore, it is worthwhile to perform a systematic review and summarize the diagnostic values of these lncRNAs.

Some meta-analyses investigated the diagnostic or prognostic value of lncRNAs. However, most of them only focused on one specific lncRNA, such as lncRNA TP73-AS1 (16), lncRNA DLX6-AS1 (17), lncRNA DRAIR (18), and lncRNA HEIH (19). Furthermore, another study used a small number of lncRNAs to determine the diagnostic value of all lncRNAs in GC but ignored the heterogeneity sample differences (20). Considering the weakness of previous studies, a more integrative meta-analysis is necessary to determine GC diagnosis *via* lncRNAs.

## Materials and methods

### Search strategy

In order to identify potentially eligible studies that were published before August 2021, two authors (JL and QX) separately conducted an electronic database search, including PubMed, Embase, and Web of science. The following search strategy was used: (Lnc RNA OR long non-coding RNA OR lncR) AND (“stomach neoplasms”[Mesh] OR “gastric cancer” OR “stomach cancer” OR “Gastric Neoplasm” OR “gastric carcinoma” OR “stomach carcinoma” OR “gastric adenocarcinoma” OR “stomach adenocarcinoma”) AND (blood OR serum OR plasma OR circulating) AND (diagnosis OR diagnostic OR diagnose).

### Literature selection

For the enrolled articles, the following inclusion criteria must be fulfilled: (1) a comparison was made between GC and healthy controls; (2) the diagnosis of GC was confirmed by a pathologist; (3) the detection technique had to be quantitative real-time PCR and test samples were from serum or plasma; and (4) sufficient data were provided to calculate 2 × 2 tables including TP (true positive), FP (false positive), TN (true negative), and FN (false negative).

The exclusion criteria were as follows: (1) duplicate articles; (2) reviews, meta-analysis, bioinformatics, case reports, and laboratory studies; (3) studies irrelevant to the diagnostic value of lncRNAs or GC; and (4) the full text was not available.

### Quality assessment

The Quality Assessment of Diagnostic Accuracy Studies 2 (QUADAS-2) (21) was applied to evaluate all enrolled articles in the meta-analysis, which mainly depend on the following domains: patient selection, index test, reference standard, and flow and timing. YZ, SB, and YD were responsible for this part of the work.

### Data extraction

Two authors (YZ and YD) independently screened the full text of every study and extracted relevant information or data including (1) basic information of the enrolled articles: the first author, publication year, country of origin, ethnicity, specimen type (serum or plasma), lncRNA type, cases, and healthy control group size, mean age, and gender distribution; and (2) sensitivity, specificity, TP, FP, FN, and TN values, which were also extracted from each article.

### Statistical methods

STATA 16.0 (Stata Corporation, College Station, TX, USA) and Revman 5.4 (The Nordic Cochrane Centre, Copenhagen, Denmark) were used to analyze extracted data. In this diagnostic meta-analysis, forest plots were applied to estimate sensitivity and specificity. The area under the curve (AUC) of the summary receiver operating curve (SROC) was used to calculate the diagnostic efficiency of serum or plasma lncRNAs in GC. According to a previous report, diagnostic efficiency can be divided into low, good, very good, and excellent in terms of AUC values:<0.75, 0.75–0.92, 0.93–0.96, and 0.97 or above (22). Meanwhile, *Q* test and Higgins *I* ^2^ statistic were used to estimate the heterogeneity among all included studies. If *I* ^2^ > 50%, signifying the existence of heterogeneity, then the random-effect model was needed for data consolidation. Otherwise, the fixed-effect model was needed. Finally, the potential bias of publication was estimated by Deeks’ funnel plot. *p* < 0.05 was considered statistically significant.

### Registration

This article has been registered on the International Platform of Registered Systematic Review and Meta-analysis Protocols (INPLASY, https://inplasy.com/); the registration number is INPLASY2022110024.

## Results

### Literature search

**Figure 1:**
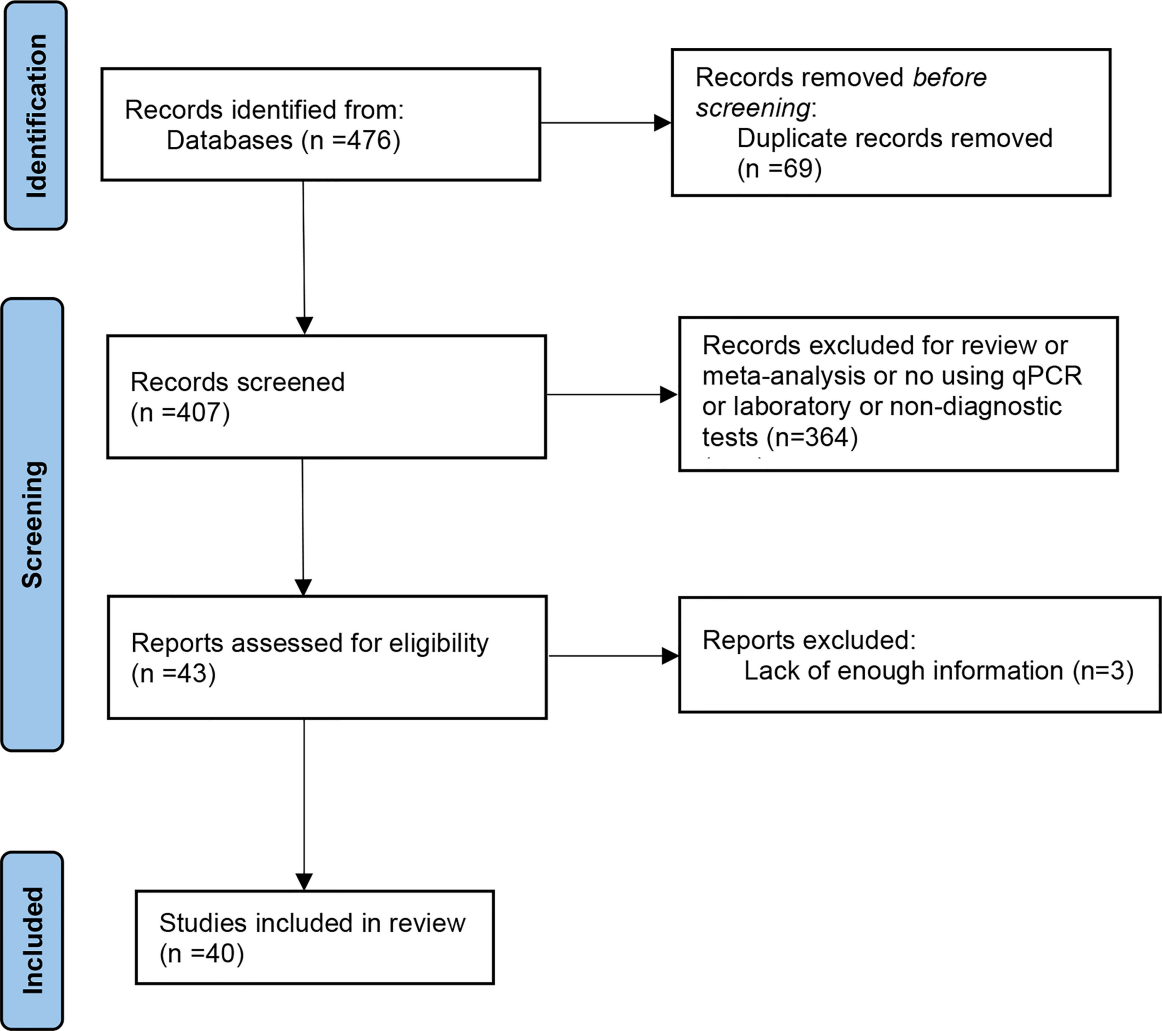
A flow diagram of the article selection process.

Through the search strategy described above, there were 476 articles from PubMed, Embase, and Web of Science included. A total of 69 duplicates were removed after a review of titles and abstracts. Next, we carefully read the rest of the articles and found 364 irrelevant publications. In addition, three articles were excluded for inadequate data. Finally, 40 publications including 6,772 participants were involved in this systematic review and meta-analysis. The basic characteristics of the included articles are listed in **Table 1**, and the flow-process diagram for the literature is presented in **Figure 1**.

**Table 1:** Characteristics of the studies included in the meta-analysis.

						Gastric cancer group			Control group				
Article ID	First author	Year	Country	Ethnicity	Total	Sample size	Mean age	Gender	Sample size	Mean age	Gender	Specimen	LncRNA
1	Shiyi Qin	2021	China	Asian	180	98	/	57/41	82	/	/	Serum	HCP5
2	Fei Han	2021	China	Asian	159	76	57.3	52/24	83	56.1	49/34	Serum	CCAT2
3	Hao Xu	2020	China	Asian	159	109	/	81/28	50	/	/	Serum	MIAT
4	Quan Zhou	2020	China	Asian	478	200	/	/	278	/	/	Serum	C5orf66-AS1
5	Hui Zhou	2020	China	Asian	159	81	64.2	51/30	78	/	/	Serum	H19
6	Peiming Zheng	2020	China	Asian	120	60	/	38/22	60	/	/	Plasma	lnc-SLC2A12-10:1
7	Guodong Zhang	2020	China	Asian	128	68	48.2	36/32	60	48.8	32/28	Plasma	PTCSC3
8	Haiyan Piao	2020	China	Asian	361	281	/	/	80	/	/	Serum	CEBPA-AS1
9	Wenwen Liu	2020	China	Asian	162	89	/	63/26	73	/	/	Serum	FEZF1-AS1, AFAP1-AS1
10	Shibao Li	2020	China	Asian	70	43	62	32/11	27	62	20/7	Serum	GNAQ-6:1
11	Rongrong Jing	2020	China	Asian	184	104	/	/	80	/	/	Serum	RP11-731F5.2
12	Wei Feng	2020	China	Asian	194	107	/	/	87	/	/	Serum	B3GALT5 AS1
13	Guohua Zhang	2019	China	Asian	106	53	/	/	53	/	/	Plasma	ARHGAP27P1
14	Ziwei Yang	2019	China	Asian	215	109	/	82/27	106	/	51/55	Plasma	PANDAR, FOXD2-AS1, SMARCC2
15	Waleed A. Mohamed	2019	Egypt	African	60	35	45.2	28/7	25	42.7	16/9	Serum	H19
16	Ying Xu	2019	China	Asian	68	34	/	/	34	/	/	Plasma	DGCR5
17	Yun Liu	2019	China	Asian	134	94	59	57/37	40	59	26/14	Serum	HOXA11-AS
18	Hong Jiang	2019	China	Asian	417	317	/	/	100	/	/	Plasma	PCGEM1
19	Bing Ji	2019	China	Asian	242	168	/	101/67	74	/	/	Plasma	LINC00086
20	Cao Peng	2019	China	Asian	160	88	47.7	52/36	72	47.1	44/28	Serum	GASL1
21	Rui Zheng	2019	China	Asian	346	173	65	111/62	173	65	110/63	Plasma	FAM49B-AS, GUSBP11, CTDHUT
22	Chenchen Cai	2019	China	Asian	92	63	/	45/18	29	/	/	Serum	PCSK2-2:1
23	Rui Zhao	2018	China	Asian	246	126	/	66/60	120	/	/	Serum	HOTTIP
24	Haipeng Xian	2018	China	Asian	100	50	61	38/12	50	61	39/11	Serum	HULC, ZNFX1-AS1
25	Xiaojie Sun	2018	China	Asian	217	117	58.33	88/29	100	49.94	58/42	Serum	CCAT2
26	Tianhang Luo	2018	China	Asian	67	46	/	/	21	/	/	Plasma	MEF2C-AS1
27	Jingjing Liu	2018	China	Asian	100	50	/	/	50	/	/	Plasma	CTC-501O10.1, AC100830.4, RP11-210K20.5
28	Eman T. Elsayed	2018	Egypt	African	100	50	/	/	50	/	/	Plasma	HOTAIR
29	Qin Lu	2017	China	Asian	152	76	63.4	50/26	76	65.4	32/44	Plasma	XIST, BCYRN1, RRP1B, TDRG1
30	Jiang Li	2017	China	Asian	180	90	66	64/26	90	60/30	64	Plasma	XIST
31	Dong Ke	2017	China	Asian	104	51	/	35/16	53	/	/	Plasma	INHBA-AS1, MIR4435-2HG, CEBPA-AS1, UCA1,AK001058
32	Yu Fan	2017	China	Asian	180	90	/	62/28	90	/	/	Serum	ANRIL
33	Lei Dong	2017	China	Asian	64	30	/	/	34	/	/	Serum	CUDR, LSINCT-5, PTENP1
34	Lin Tan	2016	China	Asian	343	263	/	/	80	/	/	Plasma	GACAT2
35	Chunjing Jin	2016	China	Asian	210	100	/	65/35	110	/	/	Serum	HULC
36	Doaa Hashad	2016	Egypt	African	62	32	43.44	19/13	30	43.53	15/15	Plasma	H19
37	Xiaoying Zhou	2015	China	Asian	140	70	/	/	70	/	/	Plasma	H19
38	Qier Li	2015	China	Asian	160	79	/	56/23	81	/	/	Plasma	LINC00152
39	Zhong Liu	2014	China	Asian	163	83	/	/	80	/	/	Plasma	FER1L4
40	Tomohiro Arita	2013	Japan	Asian	75	43	/	31/12	32	/	/	Plasma	H19

### Quality assessment

**Figure 2:**
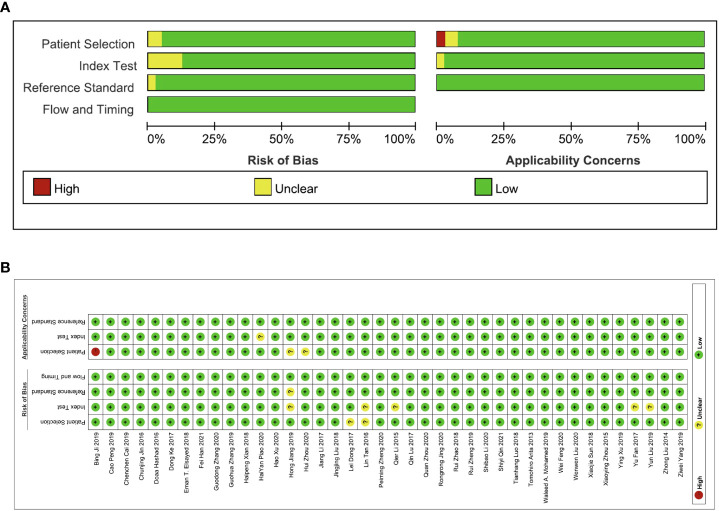
The quality assessment of the included studies *via* the QUADAS-2 tool. **(A)** Risk of bias and applicability concerns graph and **(B)** summary of quality assessment.

The QUADAS-2 tool embedded in Revman 5.4 was used to assess the quality of each study. As shown in **Figures 2A, B**, the evaluation criteria mainly focus on patient selection, index test, reference standard, and flow and timing.

### Diagnostic accuracy of circulating lncRNAs

We added all included studies to Revman 5.4, and then according to the extracted data, related figures were plotted *via* STATA 16. There were 52 lncRNAs reported among 40 studies, and their corresponding diagnostic accuracies are shown in **Table 2**. Overall sensitivity, specificity, and AUC were 0.78 (95% CI: 0.75–0.81), 0.79 (95% CI: 0.74–0.83), and 0.85 (95% CI: 0.81–0.87), respectively, which signifies a great performance for lncRNAs as noninvasive biomarkers to distinguish GC patients. The pooled diagnostic odds ratio (DOR) was 13.00 (95% CI: 10.00–17.00). Meanwhile, the pooled positive likelihood ratio (PLR) and negative likelihood ratio (NLR) were 3.70 (95% CI: 3.00–4.50) and 0.28 (95% CI: 0.24–0.32), respectively.

**Table 2:** Diagnostic accuracies of the lncRNAs mentioned in the literature.

Article ID	LncRNA	Expression	GC sample size	Control sample size	Sensitivity (%)	Specificity (%)	AUC
1	HCP5	U	98	82	0.800	0.700	0.87
2	CCAT2	U	76	83	0.8696	0.7358	0.862
3	MIAT	U	109	50	0.806	0.91	0.892
4	C5orf66-AS1	D	200	278	0.775	0.536	0.688
5	H19	U	81	78	0.7436	0.8395	0.849
6	SLC2A12-10:1	U	60	60	0.783	0.75	0.776
7	PTCSC3	D	68	60	0.897	0.846	0.92
8	CEBPA-AS1	U	281	80	0.879	0.788	0.824
9	FEZF1-AS1, AFAP1-AS1	U	89	73	0.753	0.658	0.82
10	GNAQ-6:1	D	43	27	0.837	0.556	0.736
11	RP11-731F5.2	U	104	80	0.8163	0.6364	0.78
12	B3GALT5 AS1	U	107	87	0.645	0.874	0.816
13	ARHGAP27P1	U	53	53	0.755	0.604	0.732
14	PANDAR, FOXD2-AS1, SMARCC2	U	109	106	0.797	0.846	0.839
15	H19	U	35	25	1	0.909	0.982
16	DGCR5	D	34	34	0.5939	0.8515	0.722
17	HOXA11-AS	U	94	40	0.787	0.978	0.924
18	PCGEM1	U	317	100	0.729	0.889	0.75
19	LINC00086	D	168	74	0.726	0.838	0.86
20	GASL1	D	88	72	0.841	0.81	0.8945
21	FAM49B-AS, GUSBP11, CTDHUT	U	173	173	0.775	0.739	0.818
22	PCSK2-2:1	D	63	29	0.84	0.865	0.896
23	HOTTIP	U	126	120	0.698	0.85	0.827
24	HULC, ZNFX1-AS1	U	50	50	0.58	0.8	0.85
25	CCAT2	U	117	100	0.7863	0.53	0.619
26	MEF2C-AS1	U	46	21	0.667	0.707	0.733
27	CTC-501O10.1, AC100830.4,RP11-210K20.5	D	50	50	0.99	0.49	0.764
28	HOTAIR	U	50	50	0.88	0.84	0.944
29	XIST, BCYRN1, RRP1B, TDRG1	U	76	76	0.846	0.59	0.733
30	XIST	U	90	90	0.511	0.956	0.753
31	INHBA-AS1, MIR4435-2HG, CEBPA-AS1, UCA1, AK001058	U	51	53	0.787	0.951	0.976
32	ANRIL	U	90	90	0.7444	0.889	0.83
33	CUDR, LSINCT-5, PTENP1	U	30	34	0.741	1	0.92
34	GACAT2	U	263	80	0.872	0.282	0.622
35	HULC	U	100	110	0.82	0.836	0.888
36	H19	U	32	30	0.6875	0.5667	0.724
37	H19	U	70	70	0.829	0.729	0.838
38	LINC00152	U	79	81	0.481	0.852	0.657
39	FER1L4	D	83	80	0.672	0.803	0.778
40	H19	U	43	32	0.74	0.58	0.64

### Publication bias

Deeks’ funnel plot asymmetry test was used to evaluate the publication bias of the enrolled articles. The results demonstrated a low potential for publication bias (*p* = 0.00).

## Discussion

In clinical practice, there are various noninvasive circulation biomarkers applied when screening GC patients from a healthy population. Of note, invasive diagnostic methods are unable to forecast prognosis and monitor the progress of GC. Meanwhile, the discomfort caused by such invasive tests makes it difficult for patients to accept them, thus limiting their further applications. In addition, traditional biomarkers lack enough specificity and sensitivity to diagnose GC, making their diagnostic efficacies questionable (23). Therefore, developing appropriate noninvasive biomarkers that can be used to diagnose and predict the prognosis of GC patients is of paramount importance. With the prevalence of next-generation sequencing (NGS) technology, a large number of lncRNAs have attracted tremendous attention and have been the topic of extensive research. It was revealed that lncRNAs not only participate in the transduction of various signaling pathways and thus influence cancer development (24), but also have the potential for cancer diagnosis (25, 26).

**Figure 3:**
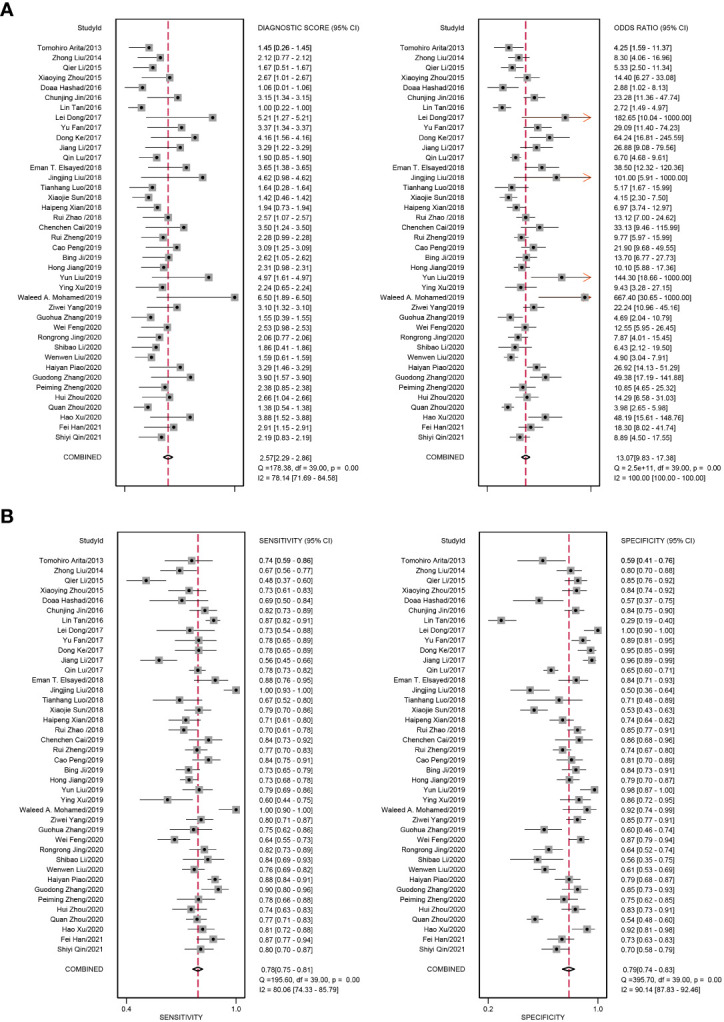
Forest plots of diagnostic accuracy of circulating lncRNAs in GC. **(A)** The pooled diagnostic score and diagnostic odds ratio (DOR) of circulating lncRNAs in the diagnosis of GC patients. **(B)** The pooled sensitivity and specificity of circulating lncRNAs in the diagnosis of GC patients.

**Figure 4:**
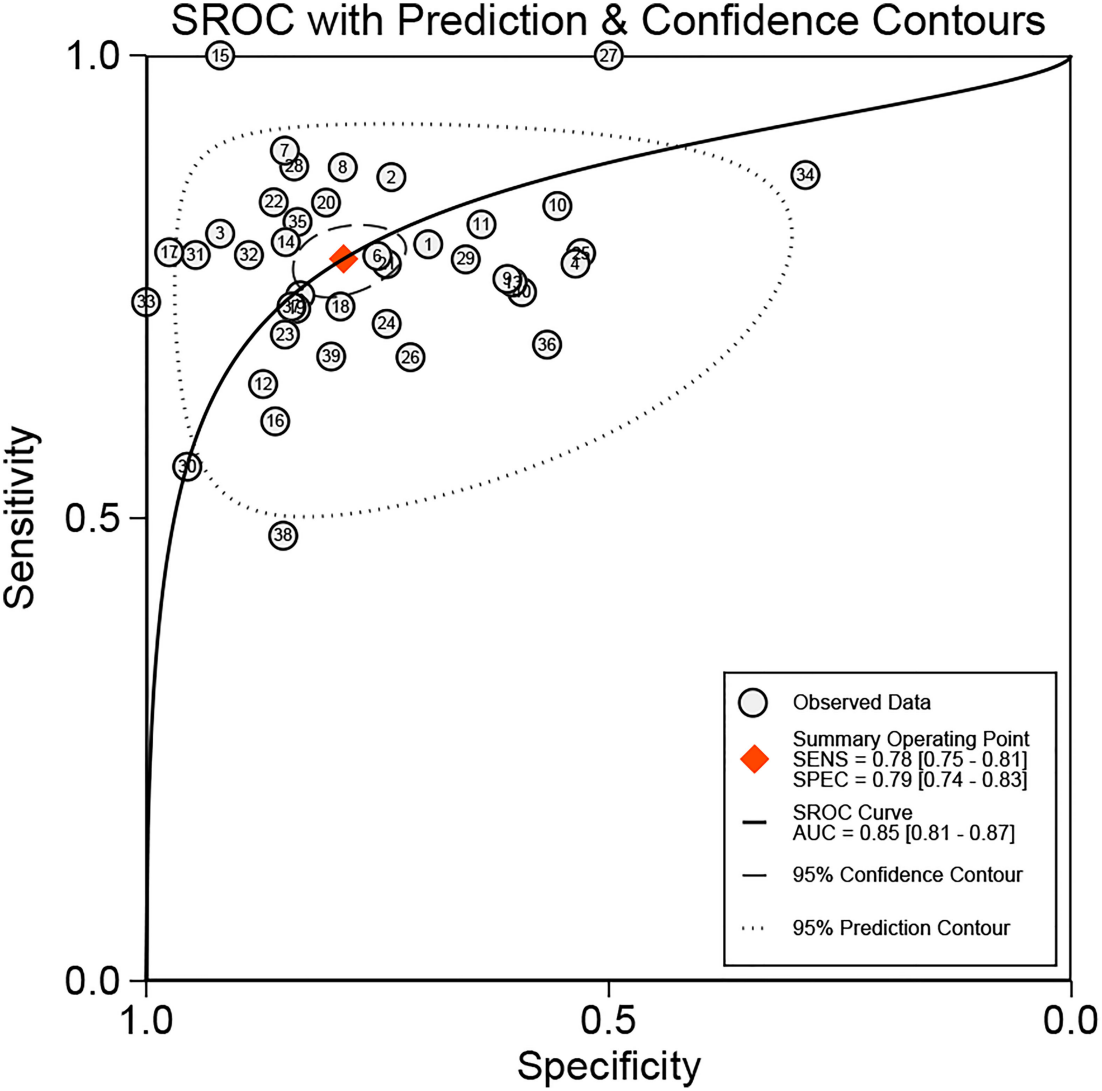
SROC of circulating lncRNAs in the diagnosis of GC patients. SROC, summary receiver operator characteristic curve; AUC, area under the curve.

**Figure 5:**
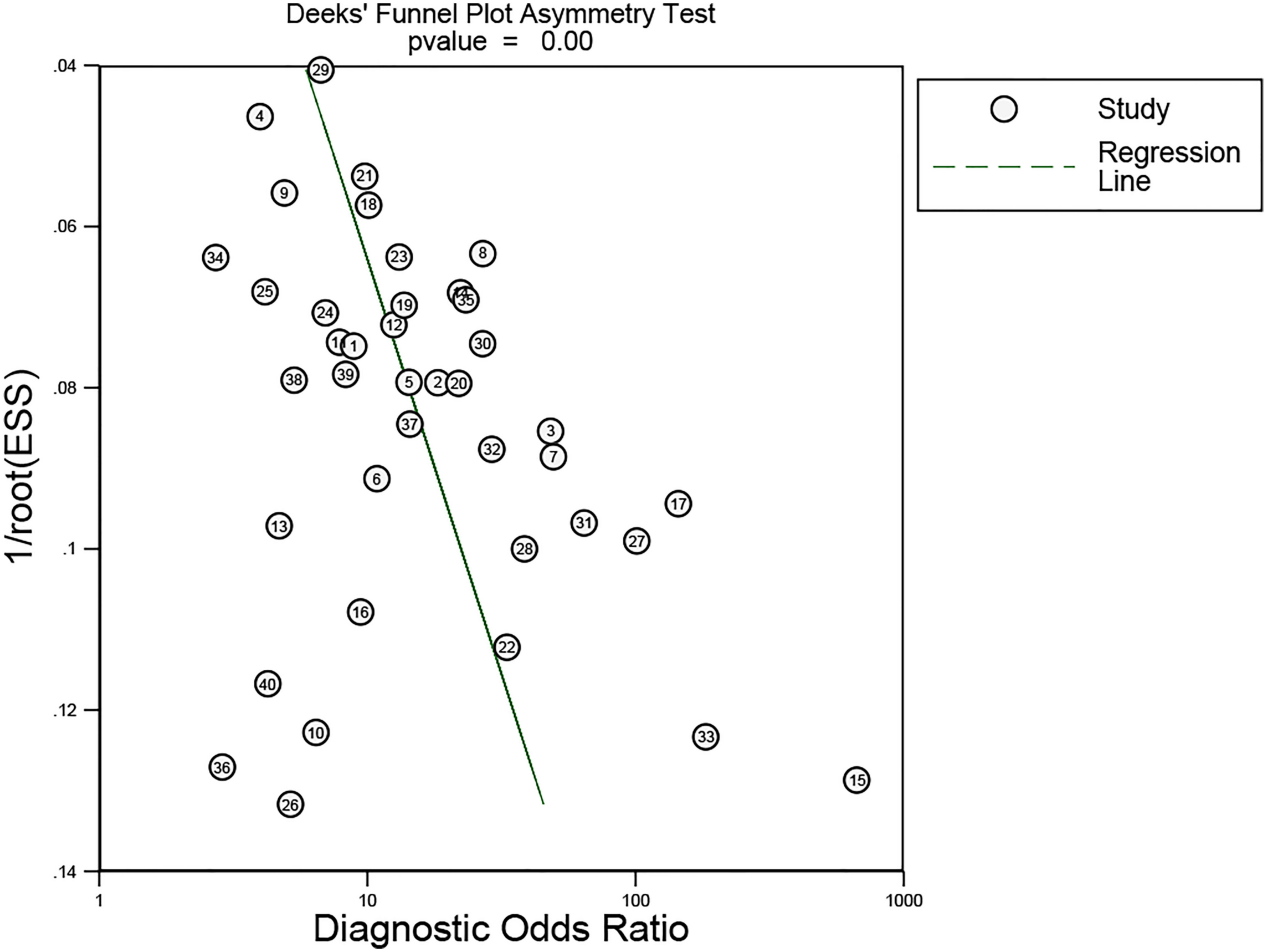
Deek’s funnel plot asymmetry test was used to estimate the publication bias for discrimination of circulating lncRNAs in GC patients.

In our meta-analysis, we included 40 original research studies including 6,772 participants to evaluate the diagnostic accuracies of lncRNAs for GC. The random-effect model was used in this meta-analysis due to the existence of heterogeneity. According to the AUC value, 5 lncRNAs with one panel of lncRNAs had a high diagnostic value, 30 lncRNAs had a moderate diagnostic value, and 4 lncRNAs had a low value. As shown in the forest plot (**Figures 3A, B**) and SROC curve (**Figure 4**), the overall sensitivity, specificity, and AUC were 0.78 (95% CI: 0.75–0.81), 0.79 (95% CI: 0.74–0.83), and 0.85 (95% CI: 0.81–0.87), respectively, which suggest that lncRNAs have a better diagnostic value than traditional tumor markers such as CEA and CA199 (27). Meanwhile, the PLR and NLR in our meta-analysis were 3.70 and 0.28, which implied that circulation lncRNAs had the ability to pick out GC patients from healthy people. As displayed in **Figure 5**, the results from Deeks’ funnel plot asymmetry test demonstrated a low potential for publication bias (*p* = 0.00). A meta-analysis enrolled 11 studies reported that circular RNAs had a high sensitivity (0.71) and specificity (0.78) as a tumor marker in the diagnosis of GC (28). Lin et al. conducted another meta-analysis to test the diagnostic potential of circRNAs in GC, and they found that the pooled sensitivity, specificity, and ROC were 0.68, 0.70, and 0.78, respectively (29). As for the microRNAs in diagnosing GC, a meta-analysis from Wei et al. revealed that circulating miRNAs also had the potential to be biomarkers in GC, which have a sensitivity of 0.76, a specificity of 0.81, and an AUC of 0.86 (30). Although the above results suggested that circRNAs and miRNAs had promising applications, we found that lncRNAs were better than them in diagnosing GC. However, the expression level of lncRNAs is a concerning issue in GC diagnosis. Depending on their role in tumor biology, not all lncRNAs are oncogenes. Some of them play a critical role in promoting tumor genesis and regulating tumor cellular properties, while others function as inhibiting factors in the development of tumors. For instance, upregulation of C5orf66-AS1 can decrease cellular activities including proliferation, migration, and invasion (31). By contrast, high expression of CCAT2 facilitates GC cell proliferation and invasion and implies poor prognosis (32). In our meta-analysis, there were 31 lncRNAs that were highly expressed and 9 lncRNAs that were downregulated in GC patients. Hence, choosing which lncRNA for early diagnosis is dependent on the actual situation and different tumors, especially when applying them as biomarkers in a clinical setting. Furthermore, more high-impact and large-scale studies are needed to illuminate the mechanism of abnormal lncRNA expression.

The research on early GC diagnosis in China began in the 1970s. With the continuous development of medical technology and the efforts of medical workers, the detection rate of early GC in China has improved, but there is still a gap compared with Japan and South Korea, because these countries have the most comprehensive GC prevention and screening programs in the world, and their early GC detection rates have reached 50% and 70% (33), respectively. There are advantages and disadvantages in diagnosing GC with lncRNAs. Traditionally, gastroscopy together with biopsy is the main method in detecting stomach lesions. However, the early diagnosis rate depends on many factors including the endoscopists’ experience and standard operation, patient cooperation during the examination, and visual clarity using endoscopy. LncRNAs are acceptable for patients because of their invasiveness. Moreover, lncRNAs are abundant in the blood. Because of their stable properties (34) and higher sensitivity and specificity than CEA and CA199, they can replace old biomarkers and, thus, can be used as auxiliary biomarkers. This study further examined the diagnostic performance of lncRNAs in GC from the perspective of a noninvasive method, which would assist with the early diagnosis of GC. Compared with previous studies (20, 35), our study had several strengths in terms of study design and data analyses. First, we included more recent eligible articles using a comprehensive and updated search strategy, which improved the precision of the estimated effect size; second, we calculated the diagnostic efficacy in one specific cancer instead of pan-cancer, which could provide more accurate supporting information in GC diagnosis; third, we performed comprehensive analyses to explore the heterogeneity and diagnostic accuracy of circulating lncRNAs in GC. The results of this study indicate that circulating lncRNAs can be used as potential biomarkers for the diagnosis of GC. There are some limitations that should not be overlooked in the present meta-analysis. First, the number of studies included is relatively small, and more studies are needed before a solid conclusion can be drawn. Second, all included studies were case–control studies instead of randomized controlled trials, which may lead to some related biases. In order to acquire high-quality evidence, more randomized controlled trials are needed to avoid biases. Third, most of the included studies were from China and most of the included patients were Asian. This could further affect the generalization of the results, which could be attributed to ethnicity differences.

Collectively, our meta-analysis revealed that serum or plasma lncRNAs have high sensitivity and specificity, which makes them clinically feasible in diagnosing GC. We believe that peripheral blood lncRNAs may become novel noninvasive biomarkers in the foreseeable future. At the same time, it should be noted that a greater number of blood samples and more evidence from rigorous multicenter clinical studies are necessary to justify their applicability as cancer biomarkers.

## Data availability statement

The original contributions presented in the study are included in the article/supplementary material. Further inquiries can be directed to the corresponding authors.

## Author contributions

Conceptualization: JL and LF. Methodology: JL, QX, YYZ, and YD. Formal analysis: JL and YQZ. Writing—original draft preparation: JL. Writing—review, and editing: JL and SB. Funding acquisition: LF. Resources: XZ. Supervision: LF and XZ. All authors contributed to the article and approved the submitted version.
